# Supplementary material for: Blood-Based Biomarkers Predictive of Metformin Target Engagement in Fragile X Syndrome
Source: Brain Sci. 2020 Jun 10;10(6):361. doi: 10.3390/brainsci10060361 (PMC7349631; doi:10.3390/brainsci10060361)
Supplement: Supplementary file 1 [file brainsci-10-00361-s001.pdf]

Supplementary Figure 1

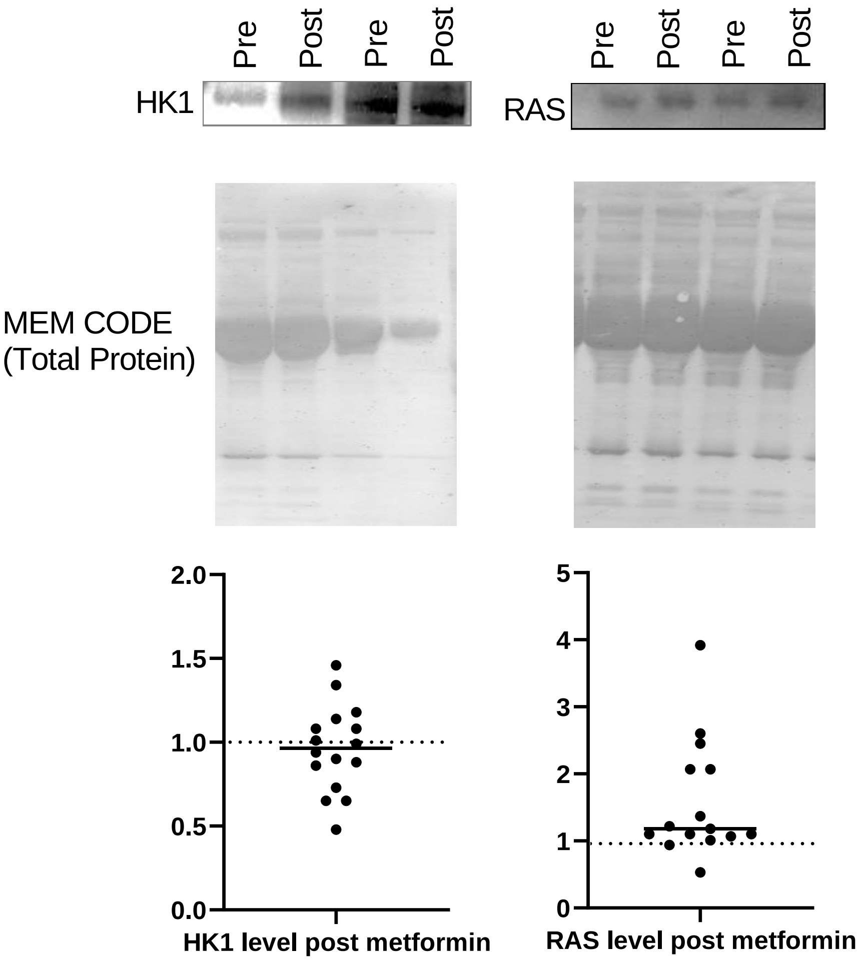

Figure S1. Exemplar blots used for protein quantification of HK1 and RAS reported in Table 2. Patient plasma was collected before and after metformin treatment then processed concurrently and run together on Western blot. Samples were blindly analyzed. Data is represented as a ratio of HK1 or RAS abundance after treatment.
